# Diagnostic accuracy of commercially available serological tests for the detection of measles and rubella viruses: a systematic review and meta-analysis

**DOI:** 10.1128/jcm.01339-23

**Published:** 2024-01-26

**Authors:** Vanessa Zubach, Jennifer Beirnes, Shannon Hayes, Alberto Severini, Joanne Hiebert

**Affiliations:** 1 Viral Exanthemata and STD Section, National Microbiology Laboratory, Public Health Agency of Canada, JC Wilt infectious Diseases Research Centre, Winnipeg, Manitoba, Canada; 2 Corporate Services Branch, Health Canada and the Public Health Agency of Canada, Government of Canada, Ottawa, Canada; 3 Department of Medical Microbiology and Infectious Diseases, Faculty of Health Sciences, University of Manitoba, Winnipeg, Manitoba, Canada; Mayo Clinic Minnesota, Rochester, Minnesota, USA

**Keywords:** meta-analysis, rubella IgM, measles IgM, systematic review

## Abstract

Measles and rubella serological diagnoses are done by IgM detection. The World Health Organization Global Measles and Rubella Laboratory Network previously endorsed Siemens Enzygnost enzyme-linked immunosorbant assay kits, which have been discontinued. A recommended replacement has not been determined. We aimed to search for suitable replacements by conducting a systematic review and meta-analysis of IgM detection methods that are currently available for measles and rubella. A systematic literature search was performed in Medline, Embase, Global Health, Cochrane Central, and Scopus on March 22 and on 27 September 2023. Studies reporting measles and/or rubella IgM detection with terms around diagnostic accuracy were included. Risk of bias was assessed using QUADAS tools. Meta-DiSc and R were used for statistical analysis. Clinical samples totalling 5,579 from 28 index tests were included in the measles meta-analysis. Sensitivity and specificity of the individual measles studies ranged from 0.50 to 1.00 and 0.53 to 1.00, respectively. Pooled sensitivity and specificity of all measles IgM detection methods were 0.94 (CI: 0.90–0.97) and 0.94 (CI: 0.91–0.97), respectively. Clinical samples totalling 4,983 from 15 index tests were included in the rubella meta-analysis. Sensitivity and specificity of the individual rubella studies ranged from 0.78 to 1.00 and 0.52 to 1.00, respectively. Pooled sensitivity and specificity of all rubella IgM detection methods were 0.97 (CI: 0.93–0.98) and 0.96 (CI: 0.93–0.98), respectively. Although more studies would be ideal, our results may provide valuable information when selecting IgM detection methods for measles and/or rubella.

## INTRODUCTION

Measles is a highly contagious, serious airborne disease caused by a virus that is known for causing rash but can also lead to severe complications and death (1). While rubella virus infection usually causes a mild fever and rash in children and adults, infection during pregnancy, especially during the first trimester, can result in miscarriage, fetal death, stillbirth, or infants with congenital malformations, known as congenital rubella syndrome (CRS) (1). These infections can be prevented with the effective live attenuated vaccine that has been available since the 1960s (2). The World Health Organization (WHO) has targeted measles and rubella for global eradication through mass immunization (3). Monitoring of progress toward global eradication and local elimination of measles and rubella requires high-quality, sensitive disease surveillance that includes laboratory confirmation of cases (4 – 6). The analysis of serum specimens for the presence of measles or rubella-specific IgM antibodies is traditionally regarded as the gold standard for laboratory confirmation (7). Common types of IgM detection methods include immunofluorescence assays (IFA), enzyme-linked immunosorbant assay (ELISA) (indirect and capture), chemiluminescent immunoassay (CLIA), and point of care tests (POCT). Previous evaluations of anti-measles and anti-rubella virus IgM detection methods resulted in the broad adoption of Enzygnost (most recently manufactured by Siemens) ELISA kits within WHO’s global measles and rubella laboratory network (8, 9). These kits have been discontinued; thus, there is an urgent need to identify replacement methods (10). To assess the diagnostic performance of commercially available IgM detection methods for measles and rubella, we performed a systematic review and meta-analysis.

## MATERIALS AND METHODS

### Study design

We adopted the Preferred Reporting Items for a Systematic review and Meta-analysis of diagnostic test accuracy (PRISMA-DTA) extension for DTA (11) guideline in preparing this report (Checklist S1 and Checklist S2 in the supplemental material).

### Literature search strategy

**Fig 1:**
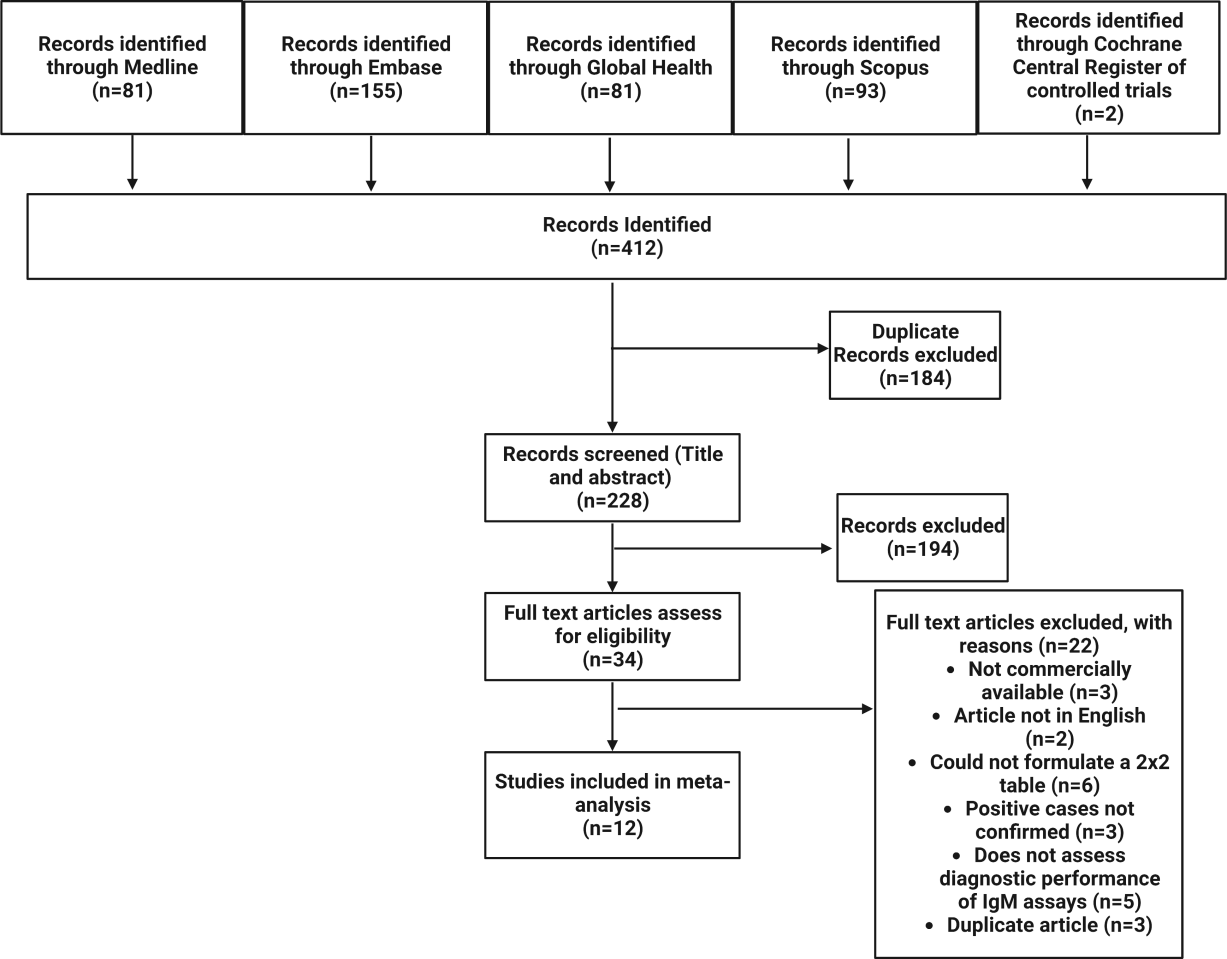
PRISMA Flow Diagram for Study Selection. Flowchart illustrating the process for the selection of the included articles for the systematic review and meta-analysis for measles and rubella IgM detection methods.

The search strategy was created by a research librarian in collaboration with members of the authorship team. A primary strategy was developed in Medline by using indexed terms and keywords pertaining to immunoglobulin M assays concerning their detection of measles and rubella. Terms around specificity, sensitivity, accuracy, and cross-reactivity were also included. Results were limited to materials published after 2013 (resulting in a 10-year period) and to only publications in English. All types of publications such as conference abstracts, commentaries, review articles, editorials, notes in addition to journal publications were included. The primary strategy was applied to Medline, Embase, Global Health, Cochrane Central, and Scopus. Searches were performed and exported on 22 March 2023 and rerun on 27 September 2023 (S1 Appendix). Additionally, reference lists of all included studies were also screened for relevant studies. The PRISMA-DTA flowchart (Fig. 1) identifying the studies included in the review was generated using Biorender (https://www.biorender.com/).

### Inclusion and exclusion criteria

Inclusion criteria in this systematic review were studies that (i) assessed the diagnostic performance for the detection of measles and or rubella IgM antibodies; (ii) used laboratory-confirmed (by virus isolation, molecular detection, and/or IgM detection methods) cases of measles or rubella in the evaluation; (iii) used either human serum or plasma as the samples; (iv) contained sufficient information to tabulate a 2 × 2 contingency table; and (v) were commercially available at the time of conducting this analysis.

### Data extraction

Based on the inclusion criteria listed above, the data extraction was done independently by two reviewers (VZ and JB). Information extracted from the articles included author information, year, study design, sample size, manufacturer, index test format, index test catalog number, reference test description, country, time of sample collection, true positive, false positive, false negative, and true negative. Any discrepancies of the extracted data were resolved by mutual agreement or by a third reviewer (JH). To conduct the analysis, samples with a borderline/equivocal result were treated as presumptive positive. Several studies evaluated more than one index test (kits under evaluation), and all the index tests data reported in each study were extracted. Entries for study design were cohort, case-control or partial cohort partial case control study. The cohort study consisted of samples that used suspected measles or rubella cases for testing. The case control study consisted of samples that used confirmed measles or rubella cases to determine the sensitivity and serum samples from healthy patients to determine the specificity. Lastly, the partial cohort partial case control study used confirmed positive specimens for measles and/or rubella (sensitivity panel) and specimens positive for other pathogens to determine the specificity.

Manufacturer data were extracted from the validation section from each of the kit inserts. When this information was not provided or published online, an email request to the manufacturer was sent for the validation data. Measles IgM ELISAs by Awareness technology, Diesse, Biorad Platelia, and Quest international were treated as the same index test; although the distributor and catalog numbers varied, the kit inserts, and the validation data were all identical leading to the conclusion that they were, in fact, the same.

A few studies (12 – 16) in our analysis contained data for the discontinued Siemens Enzygnost kits. For reader’s interest, we have included these forest plots in the supplementary data showing how the Siemens Enzygnost kits performed compared to the other index tests (Fig. S4 and S5).

### Methods for risk of bias and applicability assessment

Risk of bias and applicability. The Quality Assessment of Diagnostic Accuracy Studies 2 (QUADAS-2) (17) was used to assess the risk of bias and applicability for the studies included in the meta-analysis. Using the QUADAS-2 tool, domains investigated were patient selection, index test, reference standard, and flow and timing. An extension of the QUADAS-2 tool, the QUADAS-C tool (18) was also used to assess the risk of bias in comparative diagnostic accuracy studies. Briefly, the QUADAS-C tool was used together with the QUADAS-2 tool when a study included more than one index test. Thus, giving a separate risk of bias judgment for single index test accuracy estimates and for comparative accuracy estimates. The QUADAS-C tool assesses the risk of bias in the same four domains as the QUADAS-2 tool. The robvis visualization tool (https://www.riskofbias.info/welcome/robvis-visualization-tool) was used to generate the figures for assessing the risk of bias and applicability.

### Data analysis methods

Once the 2 × 2 contingency tables for all the index tests were tabulated, the web application Meta-DiSc 2.0 (19) was used to perform statistical analysis. For the meta-analysis of the measles and rubella studies, a univariate random effects model was used.

Using Meta-DiSc 2.0 application, forest plots of the individual index tests were prepared showing the sensitivity and specificity with corresponding 95% confidence intervals (CI). Summary Receiver Operating Curves (SROC) were generated; this approach converts each pair of sensitivity and specificity into a single measure of accuracy (20).

Where possible (studies with two or more index tests) analysis of pooled sensitivity and specificity with their corresponding 95% confidence intervals (CI), diagnostic odds ratio (DOR), and heterogeneity (*I* ^2^) results were generated with a univariate random effects model using the Meta-DiSc 2.0 application. The DOR is the odds that the test produces correct results compared to the odds of incorrect results (21). Values start at 0, and larger values indicate a better performing index test. A bivariate random effects model calculating pooled sensitivity and specificity, DOR, and heterogeneity (*I* ^2^) was also used when index tests had four or more studies. The use of the bivariate model was done for comparison with the univariate model but could not be used instead of the univariate model due to the few number of studies for many index tests. We explored heterogeneity to determine if there were notable differences between the studies. Heterogeneity was measured separately for sensitivity and specificity using the univariate random effects model in Meta-DiSc 2.0. We also confirmed these results by visually inspecting the forest plots because *I* ^2^ should be interpreted cautiously when a meta-analysis has few studies (22). We classified values as 0%–30%, low heterogeneity; 30%–50%, moderate heterogeneity; 50%–75%, substantial heterogeneity; 75%–100%, high heterogeneity. If there was substantial heterogeneity, we tried to subgroup the studies by index test format and further by index test (when there were two or more studies included). In R, the mada package v0.508, which is a tool for the meta-analysis of diagnostic accuracy studies (23), was also used to corroborate data generated by the Meta-DiSc 2.0 application.

## RESULTS

### Literature search results

The literature search strategy produced a total of 412 articles. After 184 duplicates were removed, 228 were screened at the title and abstract level. After title and abstract screening, the 34 articles remaining were further assessed. Twenty-two articles were excluded with reasons, and finally, 12 studies were subject to full text reviewing for inclusion in the meta-analysis (Fig. 1).

### Characteristics of the included studies

#### Measles

Nine of the included studies evaluated measles IgM methods, and 28 sets of data were extracted (Table 1). The breakdown of index test format was as follows: 14 were indirect ELISA, 6 were capture ELISA, and 8 were CLIA. A total of 5,579 specimens were included: 2,477 were collected from infected patients and 3,102 from non-infected patients. All studies but one had a partial cohort partial case control design. The Sowers et al. study (24) design was classified as a cohort study design because the data for the interference panel were insufficient for extracting to the 2 × 2 table. Most studies did not specify the time of sample collection post rash onset and clinical background of the study participants.

**TABLE 1:** Characteristics of the studies for the evaluation of measles IgM detection tests included in the meta-analysis^*a*^

Author (reference no.)	Year	Study design	Reference test	Index test format	Manufacturer	Catalogue number	Country
Carson (25)	2022	Partial cohort partial case-control	RT-PCR, inhouse IFA, IgM ELISA	Capture ELISA	Microimmune	MeVM010	USA
Carson (25)	2022	Partial cohort partial case-control	RT-PCR, inhouse IFA, IgM ELISA	Capture ELISA	Quest	Q01-190M	USA
Carson (25)	2022	Partial cohort partial case-control	RT-PCR, inhouse IFA, IgM ELISA	Indirect ELISA	Euroimmun	EI 2610-9601-4M	USA
Carson (25)	2022	Partial cohort partial case-control	RT-PCR, inhouse IFA, IgM ELISA	Indirect ELISA	Trinity Biotech	2326060	USA
de Ory (26)	2015	Partial cohort partial case-control	IgM ELISA	Capture CLIA	Diasorin	N/A	Spain
Gomez-Camarasa (27)	2016	Partial cohort partial case-control	Isolation and/or RT-PCR	Indirect CLIA	Vircell	N/A	Spain
Gomez-Camarasa (27)	2016	Partial cohort partial case-control	Isolation and/or RT-PCR	Capture CLIA	Diasorin	N/A	Spain
Haywood (28)	2014	Partial cohort partial case-control	IgM ELISA	Capture CLIA	Diasorin	N/A	UK
Hiebert (15)	2021	Partial cohort partial case-control	Lab and IgM serology confirmed	Indirect ELISA	Euroimmun	EI 2610-9601M	Canada
Hiebert (15)	2021	Partial cohort partial case-control	Lab and IgM serology confirmed	Indirect ELISA	Euroimmun	EI2610-9601-4M	Canada
Hiebert (15)	2021	Partial cohort partial case-control	Lab and IgM serology confirmed	Capture CLIA	Diasorin	318820	Canada
Hiebert (15)	2021	Partial cohort partial case-control	Lab and IgM serology confirmed	Capture ELISA	Microimmune	MeVM010	Canada
Hiebert (15)	2021	Partial cohort partial case-control	Lab and IgM serology confirmed	Indirect ELISA	Nova Lisa	MEAM0330	Canada
Hiebert (15)	2021	Partial cohort partial case-control	Lab and IgM serology confirmed	Indirect ELISA	Serion	ESR102M	Canada
Perez (12)	2020	Partial cohort partial case-control	IgM ELISA confirmed by consensus	Indirect ELISA	Serion	ESR102M	Spain
Perez (12)	2020	Partial cohort partial case-control	IgM ELISA confirmed by consensus	Indirect ELISA	Euroimmun	EI 2610-9601M	Spain
Perez (12)	2020	Partial cohort partial case-control	IgM ELISA confirmed by consensus	Indirect ELISA	Euroimmun	EI2610-9601-4M	Spain
Perez (12)	2020	Partial cohort partial case-control	IgM ELISA confirmed by consensus	Capture ELISA	Diesse	91073	Spain
Perez (12)	2020	Partial cohort partial case-control	IgM ELISA confirmed by consensus	Capture CLIA	Diasorin	318820	Spain
Sampedro (16)	2013	Partial cohort partial case-control	Isolation and/or RT-PCR	Capture CLIA	Diasorin	N/A	Spain
Semmler (29)	2021	Partial cohort partial case-control	RT-PCR confirmed, IgG avidity, and neutralization assays	Indirect ELISA	Euroimmun	N/A	Austria
Semmler (29)	2021	Partial cohort partial case-control	RT-PCR confirmed, IgG avidity, and neutralization assays	Capture ELISA	Biorad	N/A	Austria
Semmler (29)	2021	Partial cohort partial case-control	RT-PCR confirmed, IgG avidity, and neutralization assays	Indirect ELISA	Serion	N/A	Austria
Semmler (29)	2021	Partial cohort partial case-control	RT-PCR confirmed, IgG avidity, and neutralization assays	Capture CLIA	Diasorin	N/A	Austria
Sowers (24)	2022	Cohort	In-house capture IgM and/or RT-PCR	Indirect ELISA	Euroimmun	EL2610-9601-4M	USA
Sowers (24)	2022	Cohort	In-house capture IgM and/or RT-PCR	Indirect ELISA	Serion	ESR102M	USA
Sowers (24)	2022	Cohort	In-house capture IgM and/or RT-PCR	Capture ELISA	Awareness Technology	01-190M	USA
Sowers (24)	2022	Cohort	In-house capture IgM and/or RT-PCR	Indirect ELISA	Trinity Biotech	K140455	USA

^*a*^ N/A, not available.

#### Rubella

Four of the included studies evaluated rubella IgM methods, and 15 sets of data were tabulated (Table 2). The breakdown of index test format was as follows: eight indirect ELISA, three capture ELISA, and four CLIA. A total of 4,983 specimens were included in this study; 913 were collected from infected patients; and 4,070 from non-infected patients. All studies but one (14) had a partial cohort partial case control design. Most studies did not specify the time of sample collection post rash onset and clinical background of the study participants.

**TABLE 2:** Characteristics of the studies for the evaluation of rubella IgM detection tests included in the meta-analysis^*a*^

Author (reference no.)	Year	Study design	Reference test	Index test format	Manufacturer	Catalog number	Country
Hiebert (13)	2022	Partial cohort partial case-control	IgM ELISA confirmed	Indirect ELISA	Trinity Biotech	2325360	Canada
Hiebert (13)	2022	Partial cohort partial case-control	IgM ELISA confirmed	Indirect ELISA	Euroimmun	EI 2590-9601M	Canada
Hiebert (13)	2022	Partial cohort partial case-control	IgM ELISA confirmed	Indirect ELISA	Euroimmun	EI 2590-9601-2M	Canada
Hiebert (13)	2022	Partial cohort partial case-control	IgM ELISA confirmed	Capture ELISA	Microimmune	RuVM014	Canada
Hiebert (13)	2022	Partial cohort partial case-control	IgM ELISA confirmed	Capture ELISA	Nova Lisa	RUBM0400	Canada
Hiebert (13)	2022	Partial cohort partial case-control	IgM ELISA confirmed	Indirect ELISA	Serion	ESR129M	Canada
Hiebert (13)	2022	Partial cohort partial case-control	IgM ELISA confirmed	Capture CLIA	Diasorin	310730	Canada
Perez (12)	2020	Partial cohort partial case-control	IgM ELISA confirmed by consensus	Indirect ELISA	Serion	BC129M	Spain
Perez (12)	2020	Partial cohort partial case-control	IgM ELISA confirmed by consensus	Indirect ELISA	Euroimmun	EI 2590-9601M	Spain
Perez (12)	2020	Partial cohort partial case-control	IgM ELISA confirmed by consensus	Indirect ELISA	Euroimmun	EI 2590-9601-2M	Spain
Perez (12)	2020	Partial cohort partial case-control	IgM ELISA confirmed by consensus	Capture ELISA	Diesse	91031	Spain
Perez (12)	2020	Partial cohort partial case-control	IgM ELISA confirmed by consensus	Capture CLIA	Diasorin	310730	Spain
Perez (12)	2020	Partial cohort partial case-control	IgM ELISA confirmed by consensus	Capture CLIA	Roche	4618831190	Spain
van Helden (30)	2014	Partial cohort partial case-control	Isolation, RT-PCR, seroconversion, IgM detection, and/or IgG low avidity	Capture CLIA	Roche	N/A	Italy, France, Germany
Viswanathan (14)	2019	Cohort	IgM detection, sustained rise of rubella IgG, or RT-PCR. Consensus approach	Indirect ELISA	Euroimmun	N/A	India

^*a*^ N/A, not available.

### Studies evaluating measles IgM detection methods

**Fig 2:**
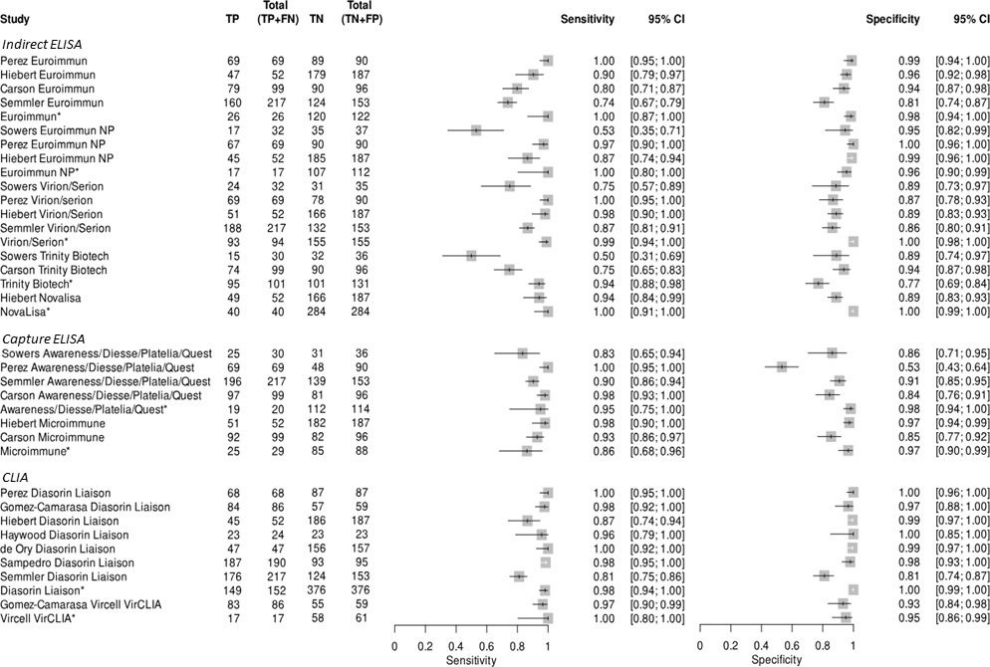
Measles IgM forest plot showing individual index test sensitivity and specificity. Index tests are grouped by test format (indirect ELISA, capture ELISA, and CLIA). Bars represent 95% confidence intervals and the boxes represent the sensitivity or specificity value. Note: TP, true positive; FN, false negative; TN, true negative; FP, false positive. *Validation data provided by manufacturer.

Forest plots for measles IgM of the calculated sensitivity and specificity were prepared (Fig. 2). Data published by the manufacturers were added to the forest plots for reference and were not included in subsequent calculations. Sensitivity and specificity of the individual studies for measles ranged from 0.50 to 1.00 and 0.53 to 1.00, respectively. Heterogeneity for sensitivity and specificity for all studies was classified as high with values of 82% and 84%, respectively, and did not improve substantially when further sub-grouped by format and index test (Table 3). This heterogeneity could be seen also in the forest plots (Fig. 2) and the SROC curves (Fig. S1).

**TABLE 3:** Measles IgM meta-analysis

Format	No. index tests	Sample size	No. infected	No. non infected	Pooled sensitivity (95% CI)	Pooled specificity (95% CI)	Diagnostic odds ratio (95% CI)	*I* ^2^ sensitivity	*I* ^2^ specificity
All	28	5,579	2,477	3,102	0.94 (0.90–0.97)	0.94 (0.91–0.97)	268.54 (122.15–590.35)	82%	84%
Indirect ELISA	14	2,765	1,141	1,624	0.90 (0.79–0.95)	0.94 (0.90–0.96)	124.23 (47.09–327.74)	80%	69%
Capture ELISA	6	1,224	566	658	0.96 (0.90–0.98)	0.87 (0.74–0.94)	144.75 (41.33–506.98)	50%	93%
CLIA	8	1,590	770	820	0.97 (0.92–0.99)	0.98 (0.94–1.00)	1,758.25 (357.85–8,638.85)	80%	82%
Euroimmun	4	963	437	526	0.91 (0.69–0.98)	0.94 (0.86–0.98)	167.99 (27.04–1,043.79)	54%	88%
Euroimmun NP	3	467	153	314	0.86 (0.56–0.97)	0.99 (0.97–1.00)	474.66 (73.47–3,066.58)	91%	25%
Virion/Serion	4	835	370	465	0.95 (0.77–0.99)	0.88 (0.84–0.90)	143.83 (22.47–920.80)	60%	0%
Trinity Biotech	2	261	129	132	0.65 (0.47–0.80)	0.92 (0.87–0.96)	22.83 (8.45–61.69)	84%	0%
Awareness / Diesse / Platelia / Quest	4	790	415	375	0.96 (0.85–0.99)	0.82 (0.65–0.91)	102.90 (19.60–540.33)	57%	93%
Microimmune	2	434	151	283	0.95 (0.90–0.97)	0.94 (0.80–0.98)	259.03 (59.81–1,121.77)	36%	91%
Diasorin Liaison	7	1,445	684	761	0.97 (0.91–0.99)	0.99 (0.94–1.00)	2,559.67 (737.19–8,887.60)	79%	84%

The pooled sensitivity and specificity for all measles index tests were 0.94 (CI 0.90–0.97) and 0.94 (CI: 0.91–0.97), respectively (Table 3). When split into subgroups according to index test format, CLIA showed the highest pooled sensitivity, pooled specificity, and diagnostic odds ratio at 0.97 (CI: 0.92–0.99), 0.98 (CI: 0.94–1.00), and 1,758.25 (CI: 357.85–8638.85), respectively. However, the heterogeneity of the studies with respect to sensitivity and specificity was still classified as high (>75%) indicating variability between studies when subgrouped by index test format. Further grouping by index test (if there were at least two studies), the Diasorin Liaison showed the highest pooled sensitivity, pooled specificity, and DOR at 0.97 (CI: 0.91–0.99), 0.99 (CI: 0.94–1.00), and 2,559.67 (CI: 737.19–8,887.60), respectively. However, the heterogeneity of both the pooled sensitivity and specificity was classified as high (>75%), likely due to a single study which did not agree with the others (Fig. 2).

Of the six ELISA tests (indirect and capture), only Euroimmun and Microimmune had both a pooled sensitivity and specificity greater than 90%. Microimmune had a higher DOR at 259.03 (CI: 59.81–1,121.77) as compared to Euroimmun at 167.99 (CI: 27.04–1,043.79). Even when studies were further subgrouped into the same index test, the heterogeneity for both the pooled sensitivity and/or specificity of all index tests was still classified as substantial or high (>50% and >75%, respectively) indicating variability between studies.

### Studies evaluating rubella IgM detection methods

**Fig 3:**
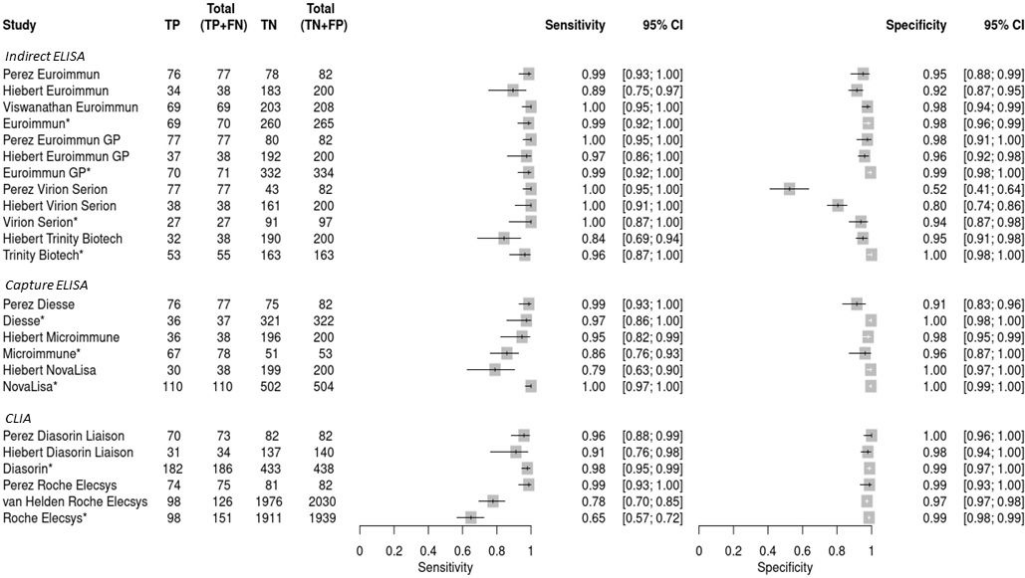
Rubella IgM forest plot showing individual index test sensitivity and specificity. Index tests are grouped by test format (indirect ELISA, capture ELISA, and CLIA). Bars represent 95% CI, and the boxes represent the sensitivity or specificity value. Note: TP, true positive; FN, false negative; TN, true negative; FP, false positive. *Validation data provided from manufacturer.

Forest plots for rubella IgM of the calculated sensitivity and specificity for all included studies were prepared (Fig. 3). Data published by the manufacturers were added for reference and not included in subsequent calculations. Sensitivity and specificity of the individual studies ranged from 0.78 to 1.00 and 0.52 to 1.00, respectively. While heterogeneity across all studies was classified as substantial or high (64% and 95% for sensitivity and specificity, respectively), there was generally less than what was seen with the measles studies within test format sub-groupings and especially when the same index test was evaluated in multiple studies (Table 4). This was also reflected in the forest plot and SROC curves (Fig. 3; Fig. S1).

**TABLE 4:** Rubella IgM meta-analysis

Format	No. index tests	Sample size	No. infected	No. non-infected	Pooled sensitivity (95% CI)	Pooled specificity (95% CI)	Diagnostic odds ratio (95% CI)	*I* ^2^ sensitivity	*I* ^2^ specificity
All	15	4,983	913	4,070	0.97 (0.93 to 0.98)	0.96 (0.93–0.98)	839.68 (273.75–2,575.54)	64%	95%
All ELISA	11	2,341	605	1,736	0.98 (0.94–0.99)	0.95 (0.89–0.98)	898.08 (216.09–3,732.43)	44%	94%
Indirect ELISA	8	1,706	452	1,254	0.99 (0.93–1.00)	0.93 (0.85–0.97)	1,314.25 (150.61–11,468.38)	10%	94%
Capture ELISA	3	635	153	482	0.94 (0.79–0.99)	0.98 (0.92–0.99)	716.28 (100.74–5,092.72)	80%	81%
CLIA	4	2,642	308	2,334	0.94 (0.82–0.98)	0.98 (0.97–0.98)	567.28 (175.57–1,832.92)	84%	0%
Euroimmun	3	674	184	490	0.98 (0.88–1.00)	0.95 (0.91–0.98)	1,089.06 (132.64–8,941.91)	46%	71%
Euroimmun GP	2	397	115	282	0.99 (0.94–1.00)	0.97 (0.94–0.98)	3,100.77 (392.36–24,504.66)	0%	0%
Virion/Serion	2	397	115	282	NE^*a*^	NE	231.88 (31.73–1,694.35)^*b*^	NE	NE
Diasorin Liaison	2	329	107	222	0.94 (0.88–0.98)	0.99 (0.96–1.00)	1,228.83 (301.27–5,012.21)	0%	0%
Roche Elecsys	2	2,313	201	2,112	0.94 (0.61–0.99)	0.97 (0.97–0.98)	537.11 (58.14–4,962.27)	89%	0%

^*a*^ NE, not estimateable, the model failed to converge on this data set.

^*b*^ The DerSimonian and Laird random effects model in R using the mada package was used to calculate this value.

The pooled sensitivity and specificity for all rubella index tests were 0.97 (CI: 0.93–0.98) and 0.96 (CI: 0.93–0.98), respectively (Table 4). ELISA methods overall had better performance than the CLIA methods but were classified as moderate and high heterogeneity (44% and 94% for sensitivity and specificity, respectively) across studies. When looking more specifically at test format, the indirect ELISA methods had the highest DOR at 1,314.25 (CI: 150.61–11,468.38). Further grouping by index test, (if there were at least two studies) improved the heterogeneity scores significantly with some exceptions (Euroimmun, Viron/Serion, and Roche Elecsys). Of the individual index tests, the Euroimmun GP ELISA had the highest pooled sensitivity, pooled specificity, and diagnostic odds ratio at 0.99 (CI: 0.94–1.00), 0.97 (CI: 0.94–0.98), and 3,100.77 (CI: 392.36–24,504.66), respectively, with heterogeneity for sensitivity and specificity classified as low among studies.

### Risk of bias and applicability

**Fig 4:**
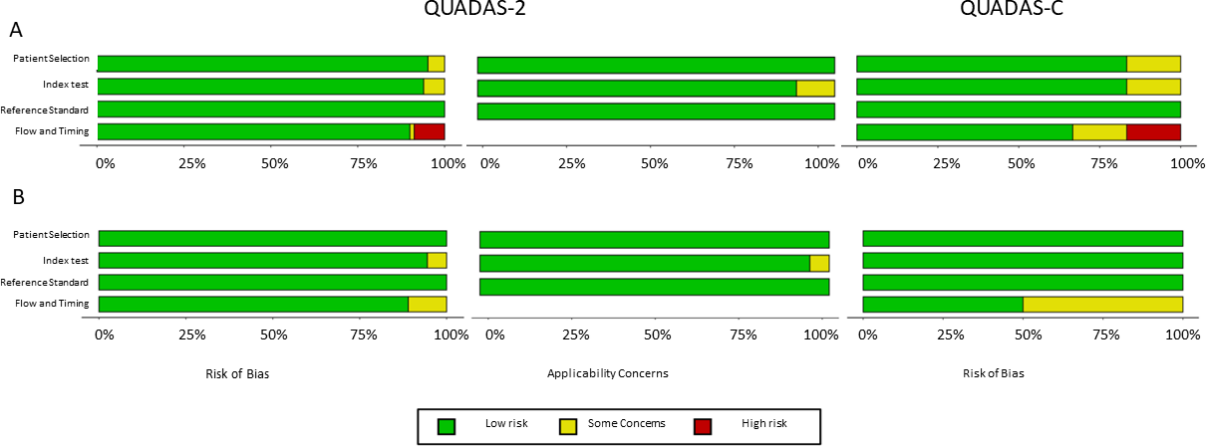
(A) Overall percentage of risk of bias and applicability concern using the QUADAS-2 and QUADAS-C tools for measles IgM detection studies. (B) Overall percentage of risk of bias and applicability concern using the QUADAS-2 and QUADAS-C tools for rubella IgM detection studies.

For the measles IgM studies, using the QUADAS-2 and QUADAS-C tools, no studies showed a high risk of bias in the patient selection, index test, or reference standard domains. For the flow and timing domain, one study (24) was given a high risk of bias because four out of five of their index tests had 2–4 samples excluded from the analysis without reason (Fig. 4A). For rubella IgM, no studies were given a high risk of bias for any of the four domains with the QUADAS-2 and QUADAS-C tools (Fig. 4B). The risk of bias and applicability concerns assessment table and individual study assessment figures can be found in the supplementary data (Tables S1 and S2; Fig. S2 and S3).

## DISCUSSION

The early and accurate diagnosis of measles and rubella is crucial to reduce their transmission. The WHO states that serological IgM detection of measles and rubella remains the gold standard for case confirmation (7). However, the lack of a WHO recommended serological assay can make it challenging for an institution to determine which kit or platform to use for detecting these viruses. This systematic review and meta-analysis was conducted to determine the pooled sensitivities, specificities, and DOR of commercially available assays for the detection of measles and rubella IgM antibodies.

From the available literature, we were able to perform meta-analysis on indirect ELISAs and capture ELISAs and CLIAs for the detection of measles and rubella IgM antibodies. For the seven measles IgM assays, pooled sensitivities and specificities were 90% or greater, for Euroimmun, MIcroimmune, and Diasorin Liaison. Overall based on our meta-analysis, the CLIA by Diasorin Liaison had the best pooled sensitivity, specificity, and DOR for the detection of measles IgM antibodies with values 0.97 (CI: 0.92–0.99), 0.98 (CI: 0.94–1.00), and 2,559.67 (CI: 737.19–8,887.60), respectively. For rubella IgM assays, pooled sensitivities and specificities were all 90% or greater and the heterogeneity for some of the rubella IgM detection assays (Euroimmun GP and Diasorin) were classified as low. Based on our meta-analysis, the indirect ELISA Euroimmun GP had the best sensitivity, specificity, and DOR for the detection of rubella IgM antibodies with values 0.99 (CI: 0.94–1.00), 0.97 (CI: 0.94–0.98), and 3,100.77 (CI: 392.36–24,504.66), respectively. Rubella IgM meta-analysis for Virion Serion data could not be computed because the univariate model used failed, but we were able to obtain the DOR using R. The individual sensitivities of the two studies including Virion Serion tests were both 1.00; however, the individual specificities deviated significantly at 0.52 and 0.80.

It is important to note that our data may appear slightly different than the original studies due to the treatment of borderline/equivocal results for index tests. In order to calculate pooled sensitivities and specificities, the results must be classified in one of two categories: positive or negative. For results classified as borderline or equivocal, we took the presumptive positive approach with the reasoning that in clinical or laboratory settings a borderline/equivocal result is usually dealt with some follow up, such as repeat testing or collecting another sample after a period of time. The proportion of borderline/equivocal results to total results in this study are 3.2% and 1.8% for measles and rubella, respectively. The inclusion of these results will not affect the general result of the meta-analysis.

Most studies used the partial cohort partial case control design to evaluate the potential for cross-reactivity of the tests, and this ability to distinguish measles and rubella infections from other pathogens is important especially in low prevalence settings. Successful surveillance efforts hinge on reliable laboratory methods that are adapted to the setting (31).

Heterogeneity is expected in any meta-analysis, and it is defined as the presence of variation, or lack of agreement, across all the studies that are grouped (32). The use of a random effects model to conduct the meta-analysis is recommended (32, 33). Sub-grouping the studies can help identify sources of heterogeneity and possibly limit their impact. In this analysis, the studies were sub-grouped by assay format and more specifically by platform, where possible. For measles, IgM methods in particular, there was still disagreement in many of the studies. Other possible sources of heterogeneity among the studies could be population factors, such as age, gender, geographic location, or vaccination history, of the individuals from whom the samples were collected. In addition, sampling time plays a key role in the detection of IgM antibodies. When serum is collected within the first few days following rash onset, a proportion of infected individuals may still test negative for IgM (7). There could also be differences in technical details in the studies such as deviations in the incubation temperatures and different lot numbers of the kits. This information was not available in most studies and could not be investigated as contributors to the heterogeneity. Information regarding the study design was available; however, most studies, except one, used the partial cohort partial case-control design, and as a result, no further investigation into study design as a source of heterogeneity could be done. Pooled sensitivity or specificity results with high heterogeneity (>75%) indicate a lack of agreement among all of the contributing studies, and therefore, the associated sensitivity or specificity metric should be interpreted with caution.

Finally, while we were interested in including evaluations of POCTs in the meta-analysis, we were only able to locate three studies of measles POCT (34 – 36) in our literature search, and they did not meet the inclusion criteria. The POCT evaluated was not commercially available, and one study was published prior to 2013. However, we have provided a forest plot in the supplementary data (Fig. S4) for interest and viewing.

Our review has some limitations. First, the few eligible studies included in the analysis limited us to using a univariate model as opposed to a bivariate model. A bivariate model has the advantage of preserving the two-dimensional (sensitivity and specificity) nature of the data, producing summary estimates of sensitivity and specificity, and acknowledging any possible (negative) correlation between these two measures (20). However, bivariate approaches cannot be recommended if the sample size is too small (33). Given the low number of studies in our meta-analysis, the use of the bivariate model was not always possible. Univariate meta-analytic methods pool sensitivity and specificity separately, ignoring any correlation that may exist between the two measures (33). When index tests had four or more studies, a bivariate random effects model was compared to data generated using the univariate data. These results between the bivariate and univariate models were comparable, see Table S3. Higher confidence in these results could have been achieved if there were more studies. There is a need for more independent research evaluating the diagnostic performance of commercial assays and platforms detecting measles and rubella IgM. For many assays, fewer than two studies were available, and as a result, meta-analysis could not be performed (includes measles: NovaLisa, VirCLIA, rubella: Trinity Biotech, Diesse, Microimmune, NovaLisa). According to our meta-analysis, several commercially available measles and rubella IgM detection assays are effective in diagnosing acute measles and rubella infections. Measles assays achieving a pooled sensitivity and specificity over 90% include Euroimmun, Microimmune, and Diasorin Liaison. Notably, the Diasorin Liaison CLIA assay achieved a pooled sensitivity and specificity greater than 95%. Rubella assays achieving a pooled sensitivity and specificity over 90% include Euroimmun, Euroimmun GP, Diasorin Liaison, and Roche Elecsys. Notably, the Euroimmun GP ELISA achieved a pooled sensitivity and specificity greater than 95%. However, the heterogeneity among the measles IgM evaluation studies and the low number of evaluations of rubella IgM detection methods highlight the need for additional independent evaluations.

## SUPPLEMENTAL MATERIAL

Checklist, appendix, and figures jcm.01339-23-s0001.pdf PRISMA-DTA checklist, S1 Appendix Search Strategy, Figures S1 - S5.

Table S1 jcm.01339-23-s0002.xlsx QUADAS-2 and QUADAS-C validation table for measles studies.

Table S2 jcm.01339-23-s0003.xlsx Table S2. QUADAS-2 and QUADAS-C validation table for rubella studies.

Table S3 jcm.01339-23-s0004.xlsx Comparison of bivariate and univariate random effects model.

ASM does not own the copyrights to Supplemental Material that may be linked to, or accessed through, an article. The authors have granted ASM a non-exclusive, world-wide license to publish the Supplemental Material files. Please contact the corresponding author directly for reuse.
