# Supplementary material for: Diagnostic accuracy of commercially available serological tests for the detection of measles and rubella viruses: a systematic review and meta-analysis
Source: J Clin Microbiol. 2024 Jan 26;62(2):e01339-23. doi: 10.1128/jcm.01339-23 (PMC10865830; doi:10.1128/jcm.01339-23)
Supplement: Checklist, appendix, and figures — PRISMA-DTA checklist, S1 Appendix Search Strategy, Figures S1 - S5. [file jcm.01339-23-s0001.pdf]

Checklist S1.

PRISMA-DTA Checklist

Section/topic	#	PRISMA-DTA Checklist Item	Reported in section
**TITLE / ABSTRACT**			
Title	1	Identify the report as a systematic review (+/- meta-analysis) of diagnostic test accuracy (DTA) studies.	Title
Abstract	2	Abstract: See PRISMA-DTA for abstracts.	S2 Checklist
**INTRODUCTION**			
Rationale	3	Describe the rationale for the review in the context of what is already known.	Abstract & Introduction
Clinical role of index test	D1	State the scientific and clinical background, including the intended use and clinical role of the index test, and if applicable, the rationale for minimally acceptable test accuracy (or minimum difference in accuracy for comparative design).	Introduction
Objectives	4	Provide an explicit statement of question(s) being addressed in terms of participants, index test(s), and target condition(s).	Introduction
**METHODS**			
Protocol and registration	5	Indicate if a review protocol exists, if and where it can be accessed (e.g., Web address), and, if available, provide registration information including registration number.	Study design
Eligibility criteria	6	Specify study characteristics (participants, setting, index test(s), reference standard(s), target condition(s), and study design) and report characteristics (e.g., years considered, language, publication status) used as criteria for eligibility, giving rationale.	Inclusion and Exclusion criteria and Data extraction
Information sources	7	Describe all information sources (e.g., databases with dates of coverage, contact with study authors to identify additional studies) in the search and date last searched.	Literature search strategy
Search	8	Present full search strategies for all electronic databases and other sources searched, including any limits used, such that they could be repeated.	Literature search strategy and S1 Appendix
Study selection	9	State the process for selecting studies (i.e., screening, eligibility, included in systematic review, and, if applicable, included in the meta-analysis).	Inclusion and Exclusion criteria
Data collection process	10	Describe method of data extraction from reports (e.g., piloted forms, independently, in duplicate) and any processes for obtaining and confirming data from investigators.	Data extraction
Definitions for data extraction	11	Provide definitions used in data extraction and classifications of target condition(s), index test(s), reference standard(s) and other characteristics (e.g. study design, clinical setting).	Data extraction
Risk of bias and applicability	12	Describe methods used for assessing risk of bias in individual studies and concerns regarding the applicability to the review question.	Quality assessment
Diagnostic accuracy measures	13	State the principal diagnostic accuracy measure(s) reported (e.g. sensitivity, specificity) and state the unit of assessment (e.g. per-patient, per-lesion).	Data analysis methods
Synthesis of results	14	Describe methods of handling data, combining results of studies and describing variability between studies. This could include, but is not limited to: a) handling of multiple definitions of target condition. b) handling of multiple thresholds of test positivity, c) handling multiple index test readers, d) handling of indeterminate test results, e) grouping and comparing tests, f) handling of different reference standards	Data analysis methods

PRISMA-DTA Checklist

Section/topic	#	PRISMA-DTA Checklist Item	Reported in section
Meta-analysis	D2	Report the statistical methods used for meta-analyses, if performed.	Data analysis methods
Additional analyses	16	Describe methods of additional analyses (e.g., sensitivity or subgroup analyses, meta-regression), if done, indicating which were pre-specified.	Data analysis methods
**RESULTS**			
Study selection	17	Provide numbers of studies screened, assessed for eligibility, included in the review (and included in meta-analysis, if applicable) with reasons for exclusions at each stage, ideally with a flow diagram.	Literature search results
Study characteristics	18	For each included study provide citations and present key characteristics including: a) participant characteristics (presentation, prior testing), b) clinical setting, c) study design, d)target condition definition, e) index test, f) reference standard, g) sample size, h) funding sources	Characteristics of studies included & Tables 1 & 2
Risk of bias and applicability	19	Present evaluation of risk of bias and concerns regarding applicability for each study.	Risk of bias & applicability, Figures 6, S1 Table & S1 Figure
Results of individual studies	20	For each analysis in each study (e.g. unique combination of index test, reference standard, and positivity threshold) report 2x2 data (TP, FP, FN, TN) with estimates of diagnostic accuracy and confidence intervals, ideally with a forest or receiver operator characteristic (ROC) plot.	Figures 2, 3, 4 and 5
Synthesis of results	21	Describe test accuracy, including variability; if meta-analysis was done, include results and confidence intervals.	Studies evaluating measles & rubella IgM detection methods Tables 3 & 4
Additional analysis	23	Give results of additional analyses, if done (e.g., sensitivity or subgroup analyses, meta-regression; analysis of index test: failure rates, proportion of inconclusive results, adverse events).	Studies evaluating measles & rubella IgM detection methods Tables 3 & 4
**DISCUSSION**			
Summary of evidence	24	Summarize the main findings including the strength of evidence.	Discussion
Limitations	25	Discuss limitations from included studies (e.g. risk of bias and concerns regarding applicability) and from the review process (e.g. incomplete retrieval of identified research).	Discussion
Conclusions	26	Provide a general interpretation of the results in the context of other evidence. Discuss implications for future research and clinical practice (e.g. the intended use and clinical role of the index test).	Discussion
**FUNDING**			
Funding	27	For the systematic review, describe the sources of funding and other support and the role of the funders.	N/A

Adapted From: McInnes MDF, Moher D, Thombs BD, McGrath TA, Bossuyt PM, The PRISMA-DTA Group (2018). Preferred Reporting Items for a Systematic Review and Meta-analysis of Diagnostic Test Accuracy Studies: The PRISMA-DTA Statement. JAMA. 2018 Jan 23;319(4):388-396. doi: 10.1001/jama.2017.19163.

For more information, visit: **www.prisma-statement.org**.

Checklist S2.

PRISMA-DTA for Abstracts Checklist

Section/topic	#	PRISMA-DTA for Abstracts Checklist item	Abstract section
**TITLE and PURPOSE**			
Title	1	Identify the report as a systematic review (+/- meta-analysis) of diagnostic test accuracy (DTA) studies.	Title
Objectives	2	Indicate the research question, including components such as participants, index test, and target conditions.	Sentences 3 & 5
**METHODS**			
Eligibility criteria	3	Include study characteristics used as criteria for eligibility.	Sentence 3
Information sources	4	List the key databases searched and the search dates.	Sentence 4
Risk of bias & applicability	5	Indicate the methods of assessing risk of bias and applicability.	Sentence 5
Synthesis of results	A1	Indicate the methods for the data synthesis.	Sentence 6
**RESULTS**			
Included studies	6	Indicate the number and type of included studies and the participants and relevant characteristics of the studies (including the reference standard).	Sentences 7 & 10
Synthesis of results	7	Include the results for the analysis of diagnostic accuracy, preferably indicating the number of studies and participants. Describe test accuracy including variability; if meta-analysis was done, include summary results and confidence intervals.	Sentences 7 to 12
**DISCUSSION**			
Strengths and limitations	9	Provide a brief summary of the strengths and limitations of the evidence	Sentence 13
Interpretation	10	Provide a general interpretation of the results and the important implications.	Sentence 13
**OTHER**			
Funding	11	Indicate the primary source of funding for the review.	N/A
Registration	12	Provide the registration number and the registry name	N/A

Adapted From: McInnes MDF, Moher D, Thombs BD, McGrath TA, Bossuyt PM, The PRISMA-DTA Group (2018). Preferred Reporting Items for a Systematic Review and Meta-analysis of Diagnostic Test Accuracy Studies: The PRISMA-DTA Statement. JAMA. 2018 Jan 23;319(4):388-396. doi: 10.1001/jama.2017.19163.

For more information, visit: **www.prisma-statement.org**.

Appendix S1 Search Strategy

Database(s): **Ovid MEDLINE(R) ALL** 1946 to March 22, 2023
Search Strategy:

#	Searches	Results
1	exp Immunoglobulin M/	55321
2	(immunoglobulin M or IgM or Ig M or immune globulin m).tw,kw,kf.	76683
3	or/1-2 [Igm]	97505
4	exp Enzyme-Linked Immunosorbent Assay/ or exp Serologic Tests/ or exp Enzyme Assays/ or exp Immunoblotting/ or Hemagglutination Inhibition Tests/ or exp Immunoassay/ or exp Immunoenzyme Techniques/ or Rapid Diagnostic Tests/	667827
5	(((enzyme-linked immunosorbent* or agglutination* or Immunofluorescen* or lateral flow* or rapid diagonstic* or rapid detection* or Immunoblot* or Hemagglutination inhibition* or Hemagglutinationinhibition* or Chemiluminescen* or flourescen* or lateral flow* or enzyme*) adj3 (techni* or test or tests or testing or analysis or analyses or assay or assays or immuno-assay* or immunoassay*)) or ELISA or LFA or RDT or IB or HAI or TPPA or CLIA or MCLIA or FIA or LFIA or EIA or LFIA).tw,kf.	437419
6	serolog*.tw,kw,kf.	132853
7	or/4-6 [Assays]	1048407
8	exp Measles/ or Measles virus/ or exp Rubella/ or "rubella virus"/	28136
9	(measle* or morbilli* or rubeola* or rubella*).tw,kw,kf.	35923
10	or/8-9 [Measles or Rubella]	40730
11	exp "Sensitivity and Specificity"/ or Cross Reactions/ or Molecular Diagnostic Techniques/ or Clinical Laboratory Techniques/	711283
12	(sensitiv* or specificit* or accurate* or accuracy or accuracies or cross react*).tw,kw,kf.	2762698
13	(((clinical* or medical) adj3 (value* or effective* or useful*)) or ((lab* or diagnostic*) adj3 (confirmation* or verification* or analysis or analyses))).tw,kf.	179097
14	or/11-13	3262904
15	3 and 7 and 10 and 14	361
16	limit 15 to yr=2013-current	86
17	limit 16 to english	81

Database(s): **Embase** 1974 to 2023 March 22
Search Strategy:

#	Searches	Results
1	immunoglobulin M/	97551
2	(immunoglobulin M or IgM or Ig M or immune globulin m).tw,kw,kf.	105464
3	or/1-2 [Igm]	138752
4	exp enzyme linked immunosorbent assay/ or exp serology/ or exp enzyme assay/ or immunoblotting/ or hemagglutination inhibition test/ or immunoassay/ or chemiluminescence immunoassay/ or enzyme immunoassay/ or fluorescence polarization immunoassay/ or immunofluorescence assay/ or measles virus test kit/ or rubella virus test kit/	928713
5	(((enzyme-linked immunosorbent* or agglutination* or Immunofluorescen* or lateral flow* or rapid diagonstic* or rapid detection* or Immunoblot* or Hemagglutination inhibition* or Hemagglutinationinhibition* or Chemiluminescen* or flourescen* or lateral flow* or enzyme*) adj3 (techni* or test or tests or testing or analysis or analyses or assay or assays or immuno-assay* or immunoassay*)) or ELISA or LFA or RDT or IB or HAI or TPPA or CLIA or MCLIA or FIA or LFIA or EIA or LFIA).tw,kf.	623892
6	serolog*.tw,kw,kf.	175249
7	or/4-6 [Assays]	1236241
8	exp measles/ or Measles virus/ or measles antibody/ or rubella antibody/	29114
9	(measle* or morbilli* or rubeola* or rubella*).tw,kw,kf.	36500
10	or/8-9 [Measles or Rubella]	44937
11	"sensitivity and specificity"/ or antibody specificity/ or cross reaction/ or molecular diagnosis/ or diagnostic accuracy/	781956
12	(sensitiv* or specificit* or accurate* or accuracy or accuracies or cross react*).tw,kw,kf.	3490622
13	(((clinical* or medical) adj3 (value* or effective* or useful*)) or ((lab* or diagnostic*) adj3 (confirmation* or verification* or analysis or analyses))).tw,kf.	254400
14	or/11-13	3949314
15	3 and 7 and 10 and 14	451
16	limit 15 to yr=2013-current	155

Database(s): **Global Health** 1973 to 2023 Week 11
Search Strategy:

#	Searches	Results
1	igm/	13905
2	(immunoglobulin M or IgM or Ig M or immune globulin m).tw.	24083
3	or/1-2 [Igm]	24083
4	elisa/ or immunoassay/ or enzyme immunoassay/ or immunological techniques/ or antibody testing/ or immunoblotting/ or immunoenzyme techniques/ or immunofluorescence/ or serology/ or immunofluorescence/ or assays/ or enzyme immunoassay/ or exp haemagglutination tests/	81055
5	(((enzyme-linked immunosorbent* or agglutination* or Immunofluorescen* or lateral flow* or rapid diagonstic* or rapid detection* or Immunoblot* or Hemagglutination inhibition* or Hemagglutinationinhibition* or Chemiluminescen* or flourescen* or lateral flow* or enzyme*) adj3 (techni* or test or tests or testing or analysis or analyses or assay or assays or immuno-assay* or immunoassay*)) or serolog* or ELISA or LFA or RDT or IB or HAI or TPPA or CLIA or MCLIA or FIA or LFIA or EIA or LFIA).tw.	186392
6	or/4-5 [Assays]	206557
7	Measles morbillivirus/ or measles/ or Rubella virus/ or rubella/	11629
8	(measle* or morbilli* or rubeola* or rubella*).tw.	15298
9	or/7-8 [Measles or Rubella]	15298
10	cross reaction/ or accuracy/ or diagnostic techniques/	101319
11	(sensitiv* or specificit* or accurate* or accuracy or accuracies or cross react*).tw.	357346
12	(((clinical* or medical) adj3 (value* or effective* or useful*)) or ((lab* or diagnostic*) adj3 (confirmation* or verification* or analysis or analyses))).tw.	23883
13	or/10-12	423948
14	3 and 6 and 9 and 13	197
15	limit 14 to yr=2013-current	93
16	limit 15 to english	81

Database(s): **EBM Reviews - Cochrane Central Register of Controlled Trials** February 2023
Search Strategy:

#	Searches	Results

1	exp Immunoglobulin M/	631
2	(immunoglobulin M or IgM or Ig M or immune globulin m).tw,kw,kf.	2581
3	or/1-2 [Igm]	2784
4	exp Enzyme-Linked Immunosorbent Assay/ or exp Serologic Tests/ or exp Enzyme Assays/ or exp Immunoblotting/ or Hemagglutination Inhibition Tests/ or exp Immunoassay/ or exp Immunoenzyme Techniques/ or Rapid Diagnostic Tests/	6506
5	(((enzyme-linked immunosorbent* or agglutination* or Immunofluorescen* or lateral flow* or rapid diagonstic* or rapid detection* or Immunoblot* or Hemagglutination inhibition* or Hemagglutinationinhibition* or Chemiluminescen* or flourescen* or lateral flow* or enzyme*) adj3 (techni* or test or tests or testing or analysis or analyses or assay or assays or immuno-assay* or immunoassay*)) or ELISA or LFA or RDT or IB or HAI or TPPA or CLIA or MCLIA or FIA or LFIA or EIA or LFIA).tw,kf.	20225
6	serolog*.tw,kw,kf.	4693
7	or/4-6 [Assays]	28632
8	exp Measles/ or Measles virus/ or exp Rubella/ or "rubella virus"/	461
9	(measle* or morbilli* or rubeola* or rubella*).tw,kw,kf.	1340
10	or/8-9 [Measles or Rubella]	1371
11	exp "Sensitivity and Specificity"/ or Cross Reactions/ or Molecular Diagnostic Techniques/ or Clinical Laboratory Techniques/	19454
12	(sensitiv* or specificit* or accurate* or accuracy or accuracies or cross react*).tw,kw,kf.	111240
13	(((clinical* or medical) adj3 (value* or effective* or useful*)) or ((lab* or diagnostic*) adj3 (confirmation* or verification* or analysis or analyses))).tw,kf.	22072
14	or/11-13	142043
15	3 and 7 and 10 and 14	4
16	limit 15 to yr=2013-current	2
17	limit 16 to english	2

Scopus

93 Results

(TITLE-ABS-KEY ("immunoglobulin M" OR igm OR "Ig M" OR "immune globulin m") AND TITLE-ABS-KEY (((("enzyme-linked immunosorbent*" OR agglutination* OR immunofluorescen* OR "lateral flow*" OR "rapid diagonstic*" OR "rapid detection*" OR

immunoblot* OR "Hemagglutination inhibition*" OR hemagglutinationinhibition* OR chemiluminescen* OR flourescen* OR "lateral flow*" OR enzyme*) W/3 (techni* OR test OR tests OR testing OR analysis OR analyses OR assay OR assays OR immuno-assay* OR immunoassay*)) OR elisa OR lfa OR rdt OR ib OR hai OR tppa OR clia OR mclia OR fia OR lfia OR eia OR lfia)) AND TITLE-ABS-KEY (measle* OR morbilli* OR rubeola* OR rubella*) AND TITLE-ABS-KEY (sensitiv* OR specificit* OR accurate* OR accuracy OR accuracies OR "cross react*" OR (((clinical* OR medical) W/3 (value* OR effective* OR useful*)) OR ((lab* OR diagnostic*) W/3 (confirmation* OR verification* OR analysis OR analyses))))) AND PUBYEAR > 2013 AND (LIMIT-TO (LANGUAGE , "English"))

Second Search

Database(s): **Ovid MEDLINE(R) ALL** 1946 to September 27, 2023
Search Strategy:

#	Searches	Results
1	exp Immunoglobulin M/	55682
2	(immunoglobulin M or IgM or Ig M or immune globulin m).tw,kw,kf.	77849
3	or/1-2 [Igm]	98687
4	exp Enzyme-Linked Immunosorbent Assay/ or exp Serologic Tests/ or exp Enzyme Assays/ or exp Immunoblotting/ or Hemagglutination Inhibition Tests/ or exp Immunoassay/ or exp Immunoenzyme Techniques/ or Rapid Diagnostic Tests/	669113
5	(((enzyme-linked immunosorbent* or enzymelinked immunosorbent* or agglutination* or Immunofluorescen* or lateral flow* or rapid diagnostic* or rapid detection* or Immunoblot* or Hemagglutination inhibition* or Hemagglutinationinhibition* or Chemiluminescen* or fluorescen* or lateral flow* or enzyme* or point* of care) adj5 (techni* or test or tests or testing or analysis or analyses or assay or assays or immuno-assay* or immunoassay*)) or ELISA? or LFA? or RDT? or IB? or HAI? or TPPA? or CLIA? or MCLIA? or FIA? or LFIA? or EIA? or LFIA? or POCT?).tw,kf.	567351
6	serolog*.tw,kw,kf.	135285
7	(NovaLisa or VirCLIA or Enzygnost or Awareness Technology or Biorad Platelia or Quest International or Siemens or Serion or Euroimmun or Trinity Biotech* or Elecsys or Diesse or Diasorin or Roche or Virion or Clin-Tech or NovaTec or Bio-Rad).tw,kw,kf.	38147
8	or/4-7 [Assays]	1201532
9	exp Measles/ or Measles virus/ or exp Rubella/ or "rubella virus"/	28267
10	(measle* or morbilli* or rubeola* or rubella*).tw,kw,kf.	36379

11	or/9-10 [Measles or Rubella]	41193
12	exp "Sensitivity and Specificity"/ or Cross Reactions/ or Molecular Diagnostic Techniques/ or Clinical Laboratory Techniques/	715818
13	(sensitiv* or specificit* or accurate* or accuracy or accuracies or cross react*).tw,kw,kf.	2850970
14	(((clinical* or medical*) adj3 (value* or effective* or useful*)) or ((lab* or diagnostic*) adj3 (confirmation* or verification* or analysis or analyses))).tw,kf.	185874
15	or/12-14	3357803
16	3 and 8 and 11 and 15	366
17	limit 16 to yr=2013-current	88
18	limit 17 to english	82

Database(s): **Embase** 1974 to 2023 September 27
Search Strategy:

#	Searches	Results
1	immunoglobulin M/	99431
2	(immunoglobulin M or IgM or Ig M or immune globulin m).tw,kw,kf.	106353
3	or/1-2 [Igm]	141121
4	exp enzyme linked immunosorbent assay/ or exp serology/ or exp enzyme assay/ or immunoblotting/ or hemagglutination inhibition test/ or immunoassay/ or chemiluminescence immunoassay/ or enzyme immunoassay/ or fluorescence polarization immunoassay/ or immunofluorescence assay/ or measles virus test kit/ or rubella virus test kit/	956763
5	(((enzyme-linked immunosorbent* or enzymelinked immunosorbent* or agglutination* or Immunofluorescen* or lateral flow* or rapid diagnostic* or rapid detection* or Immunoblot* or Hemagglutination inhibition* or Hemagglutinationinhibition* or Chemiluminescen* or fluorescen* or lateral flow* or enzyme* or point* of care) adj5 (techni* or test or tests or testing or analysis or analyses or assay or assays or immuno-assay* or immunoassay*)) or ELISA? or LFA? or RDT? or IB? or HAI? or TPPA? or CLIA? or MCLIA? or FIA? or LFIA? or EIA? or LFIA? or POCT?).tw,kf.	775184
6	serolog*.tw,kw,kf.	176812
7	(NovaLisa or VirCLIA or Enzygnost or Awareness Technology or Biorad Platelia or Quest International or Siemens or Serion or Euroimmun or Trinity Biotech* or Elecsys or Diesse or Diasorin or Roche or Virion or Clin-Tech or NovaTec or Bio-Rad).tw,kw,kf.	108515
8	or/4-7 [Assays]	1487002
9	exp measles/ or Measles virus/ or measles antibody/ or rubella antibody/	29448

10	(measle* or morbilli* or rubeola* or rubella*).tw,kw,kf.	36664
11	or/9-10 [Measles or Rubella]	45330
12	"sensitivity and specificity"/ or antibody specificity/ or cross reaction/ or molecular diagnosis/ or diagnostic accuracy/	800866
13	(sensitiv* or specificit* or accurate* or accuracy or accuracies or cross react*).tw,kw,kf.	3546961
14	(((clinical* or medical*) adj3 (value* or effective* or useful*)) or ((lab* or diagnostic*) adj3 (confirmation* or verification* or analysis or analyses))).tw,kf.	259741
15	or/12-14	4018931
16	3 and 8 and 11 and 15	458
17	limit 16 to yr=2013-current	158
18	Limit 17 to english	150

Database(s): **Global Health** 1973 to 2023 Week 38
Search Strategy:

#	Searches	Results
1	igm/	14329
2	(immunoglobulin M or IgM or Ig M or immune globulin m).tw.	24682
3	or/1-2 [Igm]	24682
4	elisa/ or immunoassay/ or enzyme immunoassay/ or immunological techniques/ or antibody testing/ or immunoblotting/ or immunoenzyme techniques/ or immunofluorescence/ or serology/ or immunofluorescence/ or assays/ or enzyme immunoassay/ or exp haemagglutination tests/	82587
5	(((enzyme-linked immunosorbent* or enzymelinked immunosorbent* or agglutination* or Immunofluorescen* or lateral flow* or rapid diagnostic* or rapid detection* or Immunoblot* or Hemagglutination inhibition* or Hemagglutinationinhibition* or Chemiluminescen* or fluorescen* or lateral flow* or enzyme* or point* of care) adj5 (techni* or test or tests or testing or analysis or analyses or assay or assays or immuno-assay* or immunoassay*)) or ELISA? or LFA? or RDT? or IB? or HAI? or TPPA? or CLIA? or MCLIA? or FIA? or LFIA? or EIA? or LFIA? or POCT?).tw.	156606
6	serolog*.tw.	89623
7	(NovaLisa or VirCLIA or Enzygnost or Awareness Technology or Biorad Platelia or Quest International or Siemens or Serion or Euroimmun or Trinity Biotech* or Elecsys or Diesse or Diasorin or Roche or Virion or Clin-Tech or NovaTec or Bio-Rad).tw.	7171
8	or/4-7 [Assays]	234400
9	Measles morbillivirus/ or measles/ or Rubella virus/ or rubella/	11845

10	(measle* or morbilli* or rubeola* or rubella*).tw.	15613
11	or/9-10 [Measles or Rubella]	15613
12	cross reaction/ or accuracy/ or diagnostic techniques/	106952
13	(sensitiv* or specificit* or accurate* or accuracy or accuracies or cross react*).tw.	369916
14	(((clinical* or medical) adj3 (value* or effective* or useful*)) or ((lab* or diagnostic*) adj3 (confirmation* or verification* or analysis or analyses))).tw.	24841
15	or/12-14	440280
16	3 and 8 and 11 and 15	203
17	limit 16 to yr=2013-current	98
18	limit 17 to english	86

Database(s): **EBM Reviews - Cochrane Central Register of Controlled Trials** August 2023
Search Strategy:

#	Searches	Results
1	exp Immunoglobulin M/	633
2	(immunoglobulin M or IgM or Ig M or immune globulin m).tw,kw,kf.	2656
3	or/1-2 [Igm]	2859
4	exp Enzyme-Linked Immunosorbent Assay/ or exp Serologic Tests/ or exp Enzyme Assays/ or exp Immunoblotting/ or Hemagglutination Inhibition Tests/ or exp Immunoassay/ or exp Immunoenzyme Techniques/ or Rapid Diagnostic Tests/	8489
5	(((enzyme-linked immunosorbent* or enzymelinked immunosorbent* or agglutination* or Immunofluorescen* or lateral flow* or rapid diagnostic* or rapid detection* or Immunoblot* or Hemagglutination inhibition* or Hemagglutinationinhibition* or Chemiluminescen* or fluorescen* or lateral flow* or enzyme* or point* of care) adj5 (techni* or test or tests or testing or analysis or analyses or assay or assays or immuno-assay* or immunoassay*)) or ELISA? or LFA? or RDT? or IB? or HAI? or TPPA? or CLIA? or MCLIA? or FIA? or LFIA? or EIA? or LFIA? or POCT?).tw.	23245
6	serolog*.tw,kw,kf.	4848
7	(NovaLisa or VirCLIA or Enzygnost or Awareness Technology or Biorad Platelia or Quest International or Siemens or Serion or Euroimmun or Trinity Biotech* or Elecsys or Diesse or Diasorin or Roche or Virion or Clin-Tech or NovaTec or Bio-Rad).tw,kw,kf.	4663
8	or/4-7 [Assays]	36590
9	exp Measles/ or Measles virus/ or exp Rubella/ or "rubella virus"/	485

10	(measle* or morbilli* or rubeola* or rubella*).tw,kw,kf.	1375
11	or/9-10 [Measles or Rubella]	1407
12	exp "Sensitivity and Specificity"/ or Cross Reactions/ or Molecular Diagnostic Techniques/ or Clinical Laboratory Techniques/	20210
13	(sensitiv* or specificit* or accurate* or accuracy or accuracies or cross react*).tw,kw,kf.	115086
14	(((clinical* or medical) adj3 (value* or effective* or useful*)) or ((lab* or diagnostic*) adj3 (confirmation* or verification* or analysis or analyses))).tw,kf.	22964
15	or/12-14	146883
16	3 and 8 and 11 and 15	4
17	limit 16 to yr=2013-current	2
18	limit 17 to english	2

108 Results

(TITLE-ABS-KEY ("immunoglobulin M" OR igm OR "Ig M" OR "immune globulin m")) AND (TITLE-ABS-KEY (((("enzyme-linked immunosorbent*" OR "enzymelinked immunosorbent*" OR agglutination* OR immunofluorescen* OR "lateral flow*" OR "rapid diagnostic*" OR "rapid detection*" OR immunoblot* OR "Hemagglutination inhibition*" OR hemagglutinationinhibition* OR chemiluminescen* OR fluorescen* OR "lateral flow*" OR enzyme* OR "point of care") W/5 (techni* OR test OR tests OR testing OR analysis OR analyses OR assay OR assays OR immuno-assay* OR immunoassay*)) OR elisa? OR lfa? OR rdt? OR ib? OR hai? OR tppa? OR clia? OR mclia? OR fia? OR lfia? OR eia? OR lfia? OR poct?))) OR (TITLE-ABS-KEY (novalisa OR virclia OR enzygnost OR "Awareness Technology" OR "Biorad Platelia" OR "Quest International" OR siemens OR serion OR euroimmun OR "Trinity Biotech*" OR elecsys OR diesse OR diasorin OR roche OR virion OR clin-tech OR novatec OR bio-rad)) AND (TITLE-ABS-KEY (measle* OR morbilli* OR rubeola* OR rubella*)) AND (TITLE-ABS-KEY (sensitiv* OR specificit* OR accurate* OR accuracy OR accuracies OR "cross react*")) OR (TITLE-ABS-KEY ((((clinical* OR medical) W/3 (value* OR effective* OR useful*)) OR ((lab* OR diagnostic*) W/3 (confirmation* OR verification* OR analysis OR analyses))))) AND PUBYEAR > 2012 AND (LIMIT-TO (LANGUAGE , "English"))

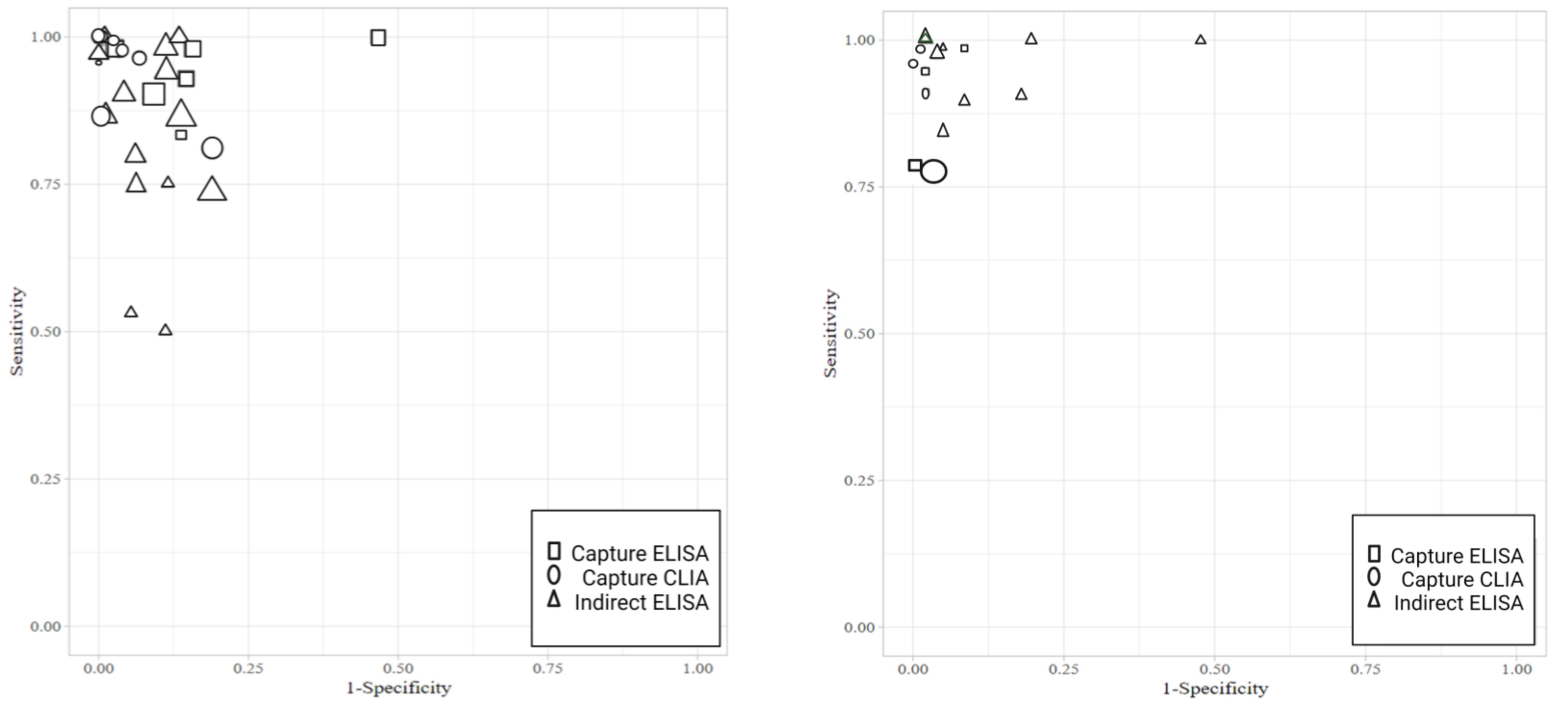

Figure S1. SROC curves for mealses IgM (right) and rubella IgM (left) of studies included in the meta-analysis. Squares indicate capture ELISA tests, circle indicate CLIA tests and triangles indicate indirect ELISAs

QUADAS-2

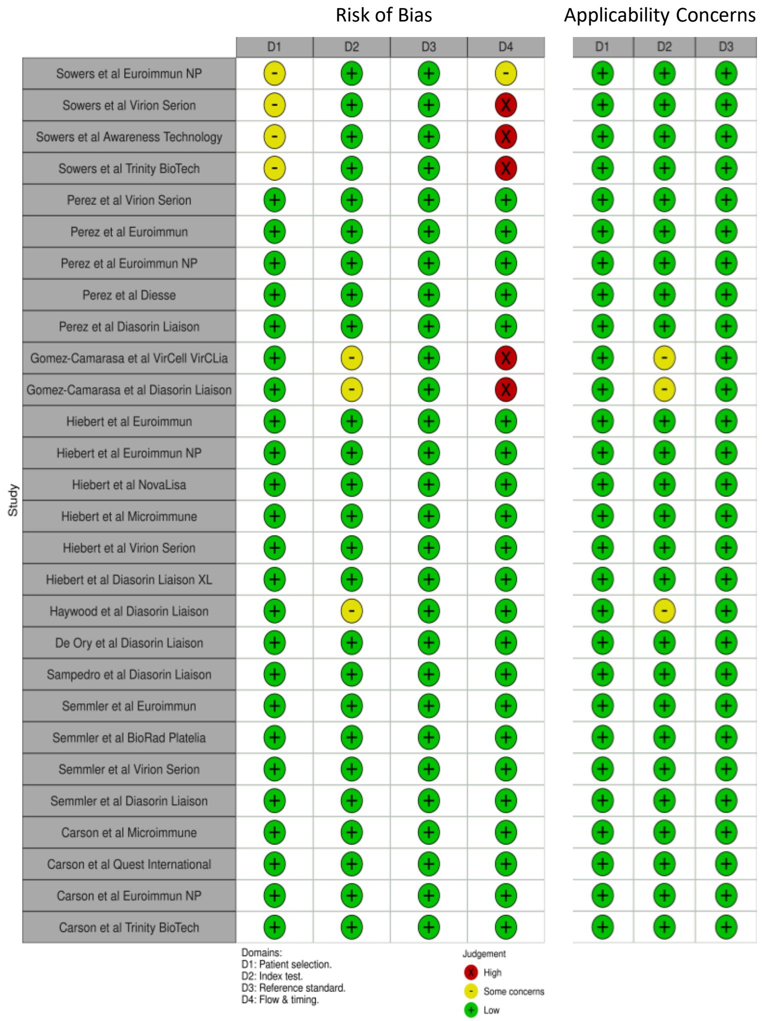

QUADAS-C

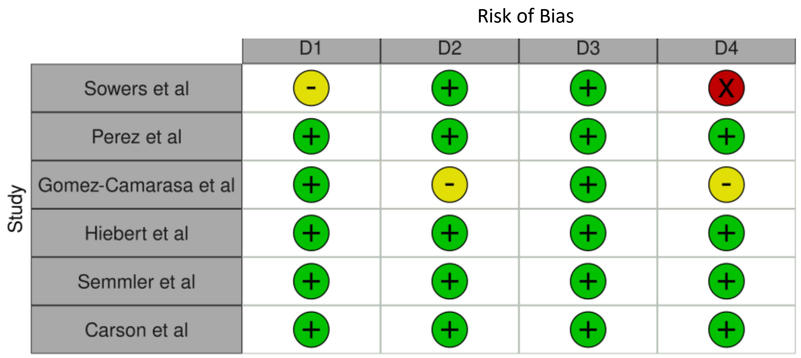

Figure S2. Risk of bias and applicability concerns assessment of individual measles studies using the Quadas-2 and Quadas-C tools.

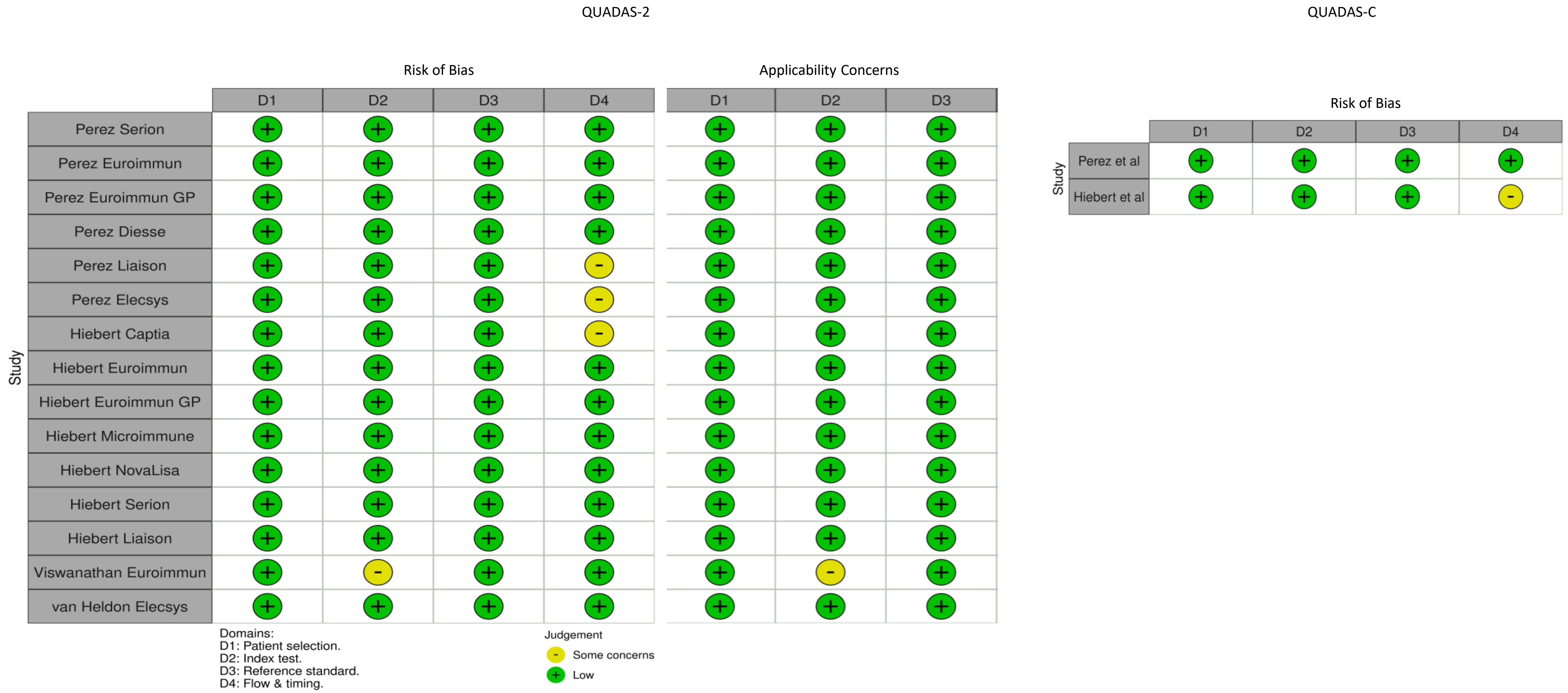

Figure S3. Risk of bias and applicability concerns assessment of individual rubella studies using the Quadas-2 and Quadas-C tools.

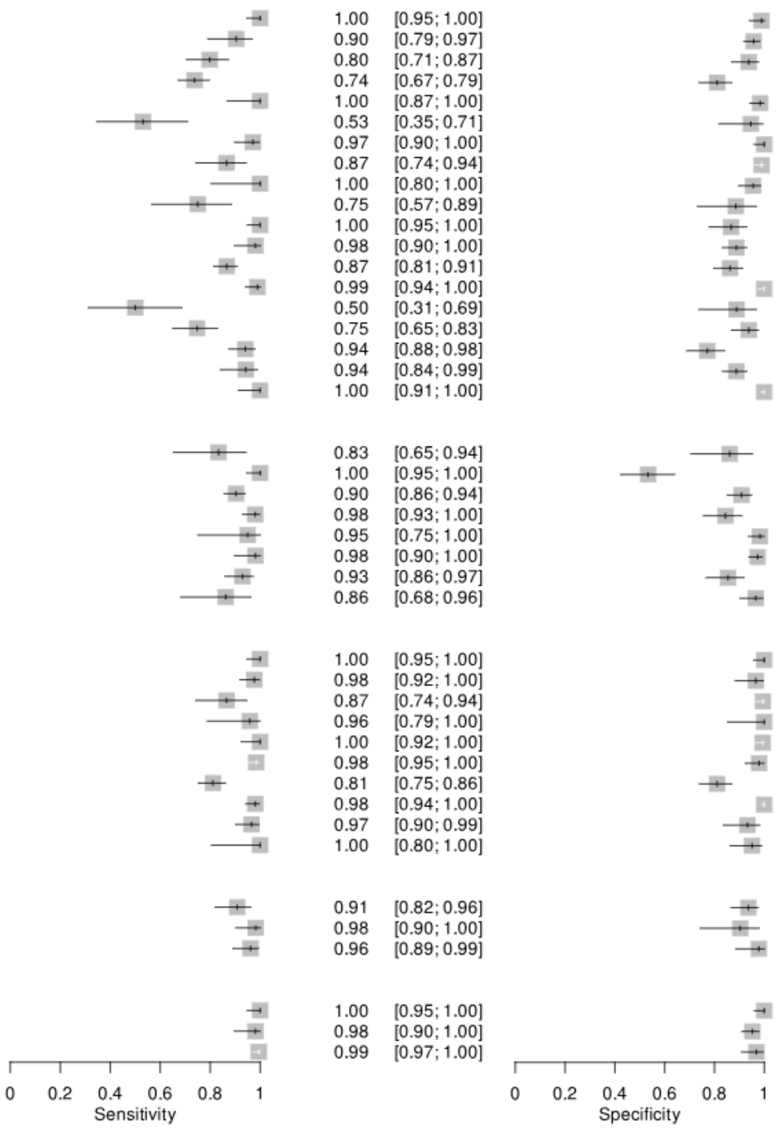

Study	TP	Total (TP+FN)	TN	Total (TN+FP)	Sensitivity	95% CI	Specificity	95% CI
Indirect ELISA								
Perez Euroimmun	69	69	89	90	1.00	[0.95; 1.00]	0.99	[0.94; 1.00]
Hiebert Euroimmun	47	52	179	187	0.90	[0.79; 0.97]	0.96	[0.92; 0.98]
Carson Euroimmun	79	99	90	96	0.80	[0.71; 0.87]	0.94	[0.87; 0.98]
Semmler Euroimmun	160	217	124	153	0.74	[0.67; 0.79]	0.81	[0.74; 0.87]
Euroimmun*	26	26	120	122	1.00	[0.87; 1.00]	0.98	[0.94; 1.00]
Sowers Euroimmun NP	17	32	35	37	0.53	[0.35; 0.71]	0.95	[0.82; 0.99]
Perez Euroimmun NP	67	69	90	90	0.97	[0.90; 1.00]	1.00	[0.96; 1.00]
Hiebert Euroimmun NP	45	52	185	187	0.87	[0.74; 0.94]	0.99	[0.96; 1.00]
Euroimmun NP*	17	17	107	112	1.00	[0.80; 1.00]	0.96	[0.90; 0.99]
Sowers Virion/Serion	24	32	31	35	0.75	[0.57; 0.89]	0.89	[0.73; 0.97]
Perez Virion/serion	69	69	78	90	1.00	[0.95; 1.00]	0.87	[0.78; 0.93]
Hiebert Virion/Serion	51	52	166	187	0.98	[0.90; 1.00]	0.89	[0.83; 0.93]
Semmler Virion/Serion	188	217	132	153	0.87	[0.81; 0.91]	0.86	[0.80; 0.91]
Virion/Serion*	93	94	155	155	0.99	[0.94; 1.00]	1.00	[0.98; 1.00]
Sowers Trinity Biotech	15	30	32	36	0.50	[0.31; 0.69]	0.89	[0.74; 0.97]
Carson Trinity Biotech	74	99	90	96	0.75	[0.65; 0.83]	0.94	[0.87; 0.98]
Trinity Biotech*	95	101	101	131	0.94	[0.88; 0.98]	0.77	[0.69; 0.84]
Hiebert Novalisa	49	52	166	187	0.94	[0.84; 0.99]	0.89	[0.83; 0.93]
NovaLisa*	40	40	284	284	1.00	[0.91; 1.00]	1.00	[0.99; 1.00]
Capture ELISA								
Sowers Awareness/Diesse/Platelia/Quest	25	30	31	36	0.83	[0.65; 0.94]	0.86	[0.71; 0.95]
Perez Awareness/Diesse/Platelia/Quest	69	69	48	90	1.00	[0.95; 1.00]	0.53	[0.43; 0.64]
Semmler Awareness/Diesse/Platelia/Quest	196	217	139	153	0.90	[0.86; 0.94]	0.91	[0.85; 0.95]
Carson Awareness/Diesse/Platelia/Quest	97	99	81	96	0.98	[0.93; 1.00]	0.84	[0.76; 0.91]
Awareness/Diesse/Platelia/Quest*	19	20	112	114	0.95	[0.75; 1.00]	0.98	[0.94; 1.00]
Hiebert Microimmune	51	52	182	187	0.98	[0.90; 1.00]	0.97	[0.94; 0.99]
Carson Microimmune	92	99	82	96	0.93	[0.86; 0.97]	0.85	[0.77; 0.92]
Microimmune*	25	29	85	88	0.86	[0.68; 0.96]	0.97	[0.90; 0.99]
CLIA								
Perez Diasorin Liaison	68	68	87	87	1.00	[0.95; 1.00]	1.00	[0.96; 1.00]
Gomez-Camarasa Diasorin Liaison	84	86	57	59	0.98	[0.92; 1.00]	0.97	[0.88; 1.00]
Hiebert Diasorin Liaison	45	52	186	187	0.87	[0.74; 0.94]	0.99	[0.97; 1.00]
Haywood Diasorin Liaison	23	24	23	23	0.96	[0.79; 1.00]	1.00	[0.85; 1.00]
de Ory Diasorin Liaison	47	47	156	157	1.00	[0.92; 1.00]	0.99	[0.97; 1.00]
Sampedro Diasorin Liaison	187	190	93	95	0.98	[0.95; 1.00]	0.98	[0.93; 1.00]
Semmler Diasorin Liaison	176	217	124	153	0.81	[0.75; 0.86]	0.81	[0.74; 0.87]
Diasorin Liaison*	149	152	376	376	0.98	[0.94; 1.00]	1.00	[0.99; 1.00]
Gomez-Camarasa Vircell VirCLIA	83	86	55	59	0.97	[0.90; 0.99]	0.93	[0.84; 0.98]
Vircell VirCLIA*	17	17	58	61	1.00	[0.80; 1.00]	0.95	[0.86; 0.99]
POCT								
Warrener 2011 et al	69	76	88	94	0.91	[0.82; 0.96]	0.94	[0.87; 0.98]
Shonhai et al 2015	55	56	28	31	0.98	[0.90; 1.00]	0.90	[0.74; 0.98]
Warrener 2023 et al	75	78	45	46	0.96	[0.89; 0.99]	0.98	[0.88; 1.00]
Siemens Enzygnost								
Perez Siemens	69	69	90	90	1.00	[0.95; 1.00]	1.00	[0.96; 1.00]
Hiebert Siemens	51	52	178	187	0.98	[0.90; 1.00]	0.95	[0.91; 0.98]
Sampedro Siemens	189	190	92	95	0.99	[0.97; 1.00]	0.97	[0.91; 0.99]

Figure S4. Forest plot for measles IgM detection methods including Siemens Enzygnost and POCT. Index tests are grouped by test format (indirect ELISA, capture ELISA, CLIA, POCT and Siemens Enzygnost). Bars represent 95% confidence intervals and the boxes represent the sensitivity or specificity value. Note: TP – true positive, FN – false negative, TN – true negative, FP – false positive. *Validation data provided from manufacturer.

Study	TP	Total (TP+FN)	TN	Total (TN+FP)	Sensitivity	95% CI	Specificity	95% CI
Indirect ELISA								
Perez Euroimmun	76	77	78	82	0.99	[0.93; 1.00]	0.95	[0.88; 0.99]
Hiebert Euroimmun	34	38	183	200	0.89	[0.75; 0.97]	0.92	[0.87; 0.95]
Viswanathan Euroimmun	69	69	203	208	1.00	[0.95; 1.00]	0.98	[0.94; 0.99]
Euroimmun*	69	70	260	265	0.99	[0.92; 1.00]	0.98	[0.96; 0.99]
Perez Euroimmun GP	77	77	80	82	1.00	[0.95; 1.00]	0.98	[0.91; 1.00]
Hiebert Euroimmun GP	37	38	192	200	0.97	[0.86; 1.00]	0.96	[0.92; 0.98]
Euroimmun GP*	70	71	332	334	0.99	[0.92; 1.00]	0.99	[0.98; 1.00]
Perez Virion Serion	77	77	43	82	1.00	[0.95; 1.00]	0.52	[0.41; 0.64]
Hiebert Virion Serion	38	38	161	200	1.00	[0.91; 1.00]	0.80	[0.74; 0.86]
Virion Serion*	27	27	91	97	1.00	[0.87; 1.00]	0.94	[0.87; 0.98]
Hiebert Trinity Biotech	32	38	190	200	0.84	[0.69; 0.94]	0.95	[0.91; 0.98]
Trinity Biotech*	53	55	163	163	0.96	[0.87; 1.00]	1.00	[0.98; 1.00]
Capture ELISA								
Perez Diesse	76	77	75	82	0.99	[0.93; 1.00]	0.91	[0.83; 0.96]
Diesse*	36	37	321	322	0.97	[0.86; 1.00]	1.00	[0.98; 1.00]
Hiebert Microimmune	36	38	196	200	0.95	[0.82; 0.99]	0.98	[0.95; 0.99]
Microimmune*	67	78	51	53	0.86	[0.76; 0.93]	0.96	[0.87; 1.00]
Hiebert NovaLisa	30	38	199	200	0.79	[0.63; 0.90]	1.00	[0.97; 1.00]
NovaLisa*	110	110	502	504	1.00	[0.97; 1.00]	1.00	[0.99; 1.00]
Indirect ELISA								
Perez Diasorin Liaison	70	73	82	82	0.96	[0.88; 0.99]	1.00	[0.96; 1.00]
Hiebert Diasorin Liaison	31	34	137	140	0.91	[0.76; 0.98]	0.98	[0.94; 1.00]
Diasorin*	182	186	433	438	0.98	[0.95; 0.99]	0.99	[0.97; 1.00]
Perez Roche Elecsys	74	75	81	82	0.99	[0.93; 1.00]	0.99	[0.93; 1.00]
van Helden Roche Elecsys	98	126	1976	2030	0.78	[0.70; 0.85]	0.97	[0.97; 0.98]
Roche Elecsys*	98	151	1911	1939	0.65	[0.57; 0.72]	0.99	[0.98; 0.99]
Siemens Enzygnost								
Perez Siemens	77	77	72	82	1.00	[0.95; 1.00]	0.88	[0.79; 0.94]
Hiebert Siemens	38	38	179	200	1.00	[0.91; 1.00]	0.90	[0.84; 0.93]
Viswanathan Siemens	65	69	208	208	0.94	[0.86; 0.98]	1.00	[0.98; 1.00]

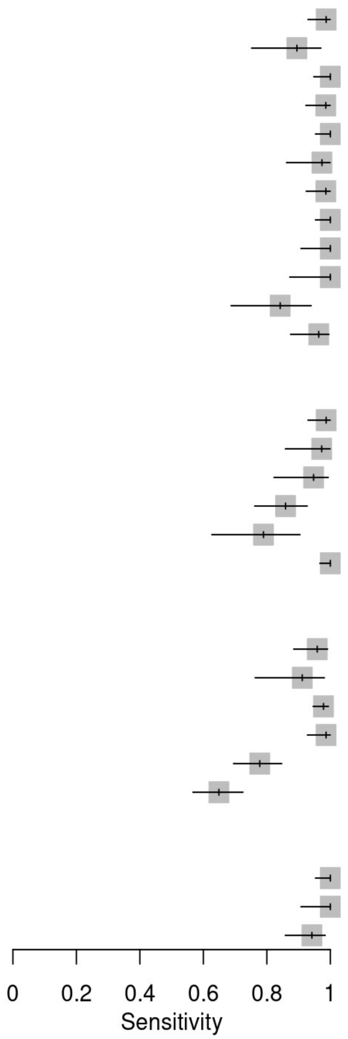

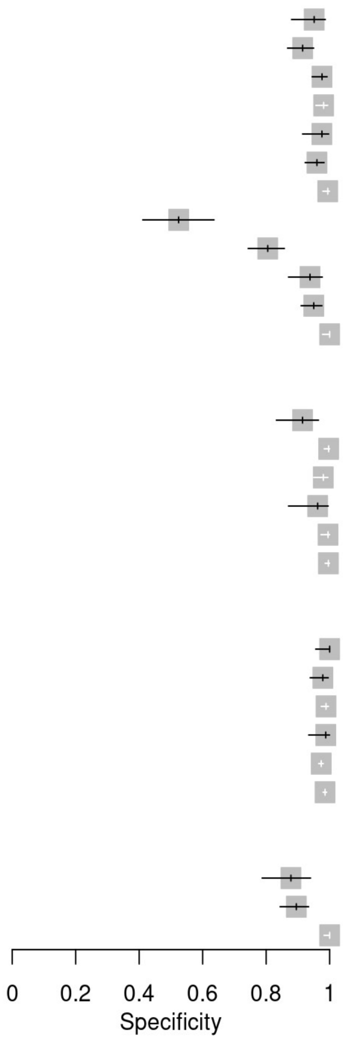

Figure S5. Forest plot for rubella IgM detection methods including Siemens Enzygnost. Index tests are grouped by test format (indirect ELISA, capture ELISA, CLIA and Siemens Enzygnost). Bars represent 95% confidence intervals and the boxes represent the sensitivity or specificity value. Note: TP – true positive, FN – false negative, TN – true negative, FP – false positive. *Validation data provided from manufacturer.
